# Biology

**DOI:** 10.1080/13577140120097111

**Published:** 2001-11


### BIOLOGY

### 032 Is Cytogenetic Analysis Clinically Useful? An Analysis of 101 Consecutive Cases of Benign and Malignant Bone and Soft Tissue Tumors of the Extremities

Robert M Henshaw[1], Barry M Shmookler[2], Martin M Malawer[1]
*[1]Department of Orthopedic Oncology Washington Cancer Institute Washington Hospital Center, [2]Department of Pathology Suburban Hospital*

*Objective:* The purpose of this study was to evaluate the results and clinical usefulness of cytogenetic analysis when routinely performed for bone and soft tissue tumors.
*Methods:* 101 (51 malignant/50 benign) consecutive musculoskeletal tumors surgically excised at our institution underwent both cytogenetic analysis and traditional histologic evaluation. The successful culture rate for the cytogenetic analysis was 86%. 57% (26/46) of clearly malignant tumors successfully cultured demonstrated significant clonal abnormalities. 46% (19/41) of benign tumors cultured had significant cytogenetic clonal aberrations, including 8 lipomas, 4 PVNS, 3 GCT, 2 fibromatosis, 1 chondroblastoma and 1 schwannoma.
*Results:* Specific cytogenetic aberrations seen in various benign tumors included chromosomal deletions, trisomies, translocations, inversions, ring and marker formations, as well as dicentric and telomeric associations. Increased cellular ploidy (more than 50 chromosomes per cell) was demonstrated in 16/46 malignant and 1/41 benign tumors. Hyperploidy was highly correlated with malignancy ($p<0.0004$, chi squared analysis): the only "benign" tumor was a multiply recurrent GCT demonstrating histologic changes consistent with early sarcomatous transformation. As expected, cytogenetic abnormalities frequently occurred in malignant tumors. Surprisingly, almost half of the benign tumors tested had significant clonal cytogenetic aberrations. Consistent findings of extra chromosomes 5 and 7 in samples of PVNS strongly favor a neoplastic origin for this condition.
*Conclusion:* Although the presence or absence of cytogenetic aberrations cannot be used as a determinant of malignant potential, increased cellular ploidy is highly indicative of malignancy. Cytogenetic analysis can be useful in classifying the malignant potential of recurrent and difficult to diagnose tumors of the musculoskeletal system.

### 035 Molecular Genetic Characterization of Fusion Genes in Extraskeletal Myxoid Chondrosarcomas

I Panagopoulos[1], F Mertens[1], N Mandahl[1], O Brosjö[2], S Heim[3], B Bjerkehagen[3], R Sciot[4], P Dal Cin[5], J A Fletcher[5], C D Fletcher[5]
*[1]Dept of Clinical Genetics, Lund University, [2]Dept of Orthopedics, Karolinska Hospital, [3]The Norwegian Radium Hospital, [4]Dept of Pathology, [5]Dept of Pathology, Brigham and Women's Hospital*

*Objective:* Extraskeletal myxoid chondrosarcoma (EMC) is a soft tissue neoplasm cytogenetically characterized by t(9;22)(q22;q11-12), generating a hybrid EWS/CHN gene, or a t(9;17)(q22;q11), resulting in an RBP56/CHN chimera. Prior to the present study, only 24 EMCs had been analyzed for the fusion transcripts, and the expression of the native CHN gene had been examined in only two cases. The aim of the present study was to characterize the fusion transcript in 18 cases, and to correlate the findings with karyotypic data.
*Methods:* The study was based on 18 surgical biopsies (17 from primary lesions, one from a metastasis) from 18 patients (15 men, three women; age at diagnosis 35–79 years) with EMC RT-PCR was carried out for the detection of the EWS/CHN and RBP56/CHN chimeric transcripts as well as for the expression of the full length transcript of CHN and the two additional CHN transcript variants: the 3′-end truncated mRNA form and the Nor1b variant with the alternative 5′-untranslated region. Cytogenetic analysis was performed after short-term culturing.
*Results:* Chromosomal aberrations were detected in 16/17 cases in our series; 13 with involvement of 9q22 and 22q12, and three with rearrangements of 9q22 and 17q11. Fifteen cases carried an EWS/CHN fusion transcript and three an RBP56/CHN transcript. The most frequent EWS/CHN transcript (10 tumors), was fusion of exon 12 of EWS with exon 3 of CHN (type 1), followed by fusion of exon 13 of EWS with exon 3 of CHN (two cases; type 5). In tumors with RBP56/CHN fusion, exon 6 of RBP56 was fused to exon 3 of CHN. RT-PCR analysis of the native CHN showed that it was expressed in 11 tumors, nine of which expressed both the long and short 3′ terminals, with and without the ligand domain, respectively.
*Conclusion:* From the distribution of secondary chromosomal abnormalities and previous in vitro studies of the EWS and RBP56 genes, one might predict that type of chimeric transcript is of biologic significance. Indeed, in the present study, all four patients who developed metastases had tumors with EWS/CHN fusions.

### 042 Gene Expression in Lipoma, Hibernoma, and Liposarcoma

Keith M Skubitz, Edward C Cheng, Denis R Clohisy, Roby C Thompson, Carlos J Manivel, Amy P Skubitz
*University of Minnesota*

*Objective:* Malignant transformation is thought to be associated with changes in the expression of a number of genes, and this alteration in gene expression is felt to be critical to the development of the malignant phenotype. In many cases, the progression to malignant transformation is associated with the sequential acquisition of multiple mutations. Sarcomas represent a diverse group of tumors felt to be derived from cells of mesenchymal origin. Marked heterogeneity exists in the biological behavior of sarcomas, even within histologic subtypes of sarcomas. In an effort to better understand the biology of sarcomas, we are examining gene expression using the Affymetrix microarray technology.
*Methods:* In this study, the expression of ~60,000 genes/ESTs in lipomas, hibernomas, intra-muscular lipomas, and liposarcomas was determined by Gene Logic. Differences in gene expression were quantified as the fold change in gene expression in lipomas compared with hibernoma, intra-muscular lipoma, atypical lipomatous tumor, and liposarcoma.
*Results:* Nine genes were expressed greater than 20 fold (1 greater than 70 fold) more in lipomas than in lipomatosis, and 4 were expressed greater than 20 fold more in lipomatosis. Twelve genes were expressed greater than 20 fold (3 greater than 80 fold) more in lipoma than in hibernoma, and 13 were expressed greater than 20 fold more in hibernoma. Interestingly, the thyroid hormone responsive "spot 14" was more highly expressed in lipoma. Eight genes were expressed greater than 50 fold (3 greater than 80 fold) more in lipoma than in intra-muscular lipoma. Seven genes were expressed greater than 20 fold more in lipoma than in liposarcoma, and 1 was expressed greater than 20 fold more in liposarcoma.
*Conclusion:* We conclude that differences in gene expression may help differentiate among subtypes of sarcomas, and may also yield clues to the pathophysiology of this heterogeneous group of tumors.

DOI: 10.1080/13577140120097111

**051 High Quality RNA Isolation from Chondrosarcoma; Application to cDNA Microarrays**

H. J. Baelde[1], A. M. Cleton-Jansen[1], H. van Beerendonk[1], M. Namba[2], J. V. Bovee[1], P. C. Hogendoorn[1]
*[1]Department of Pathology, Leiden University Medical Center, [2]Department of Cell Biology, Institute of Molecular and Cellular Biology, Okayama University Medical School*

*Objective:* High quality RNA isolation from cartilaginous tissue is considered difficult due to relative low cellularity and the abundance of extracellular matrix rich in glycosaminoglycans and collagen. Given the growing interest and technical possibilities to study RNA expression at a high throughput level research on certain tumour types is hampered because of aforementioned characteristics.
*Methods:* We present a robust method using a combination of two RNA isolation procedures that has been developed to obtain high molecular weight RNA from fresh frozen and stored tissue of normal cartilage and cartilaginous tumors. Using this method RNA was isolated from normal cartilage, peripheral and central chondrosarcoma and from chondrosarcoma cell lines SW1353 and OUMS-27. RNA quality was validated after electrophoresis and staining with ethidium bromide. Subsequent conversion to cDNA and labelling was followed by application to a Micromax Human cDNA microarray system and fluorescence intensity was scanned using an Affimetrix/GMS 418 scanner and analysed with a Gene-Pix Pro 3.0 analysis program.
*Results:* The yields range from 0.1-0.5 mg RNA per mg tissue. RNA samples from normal cartilage and from two chondrosarcomas isolated using this method were applied successfully in cDNA microarray experiments. The number of genes that give interpretable results is in the range of what is expected when compared with microarray results obtained on chondrosarcoma cell line RNA. Signal-to-noise ratios are good and differential expression between tumor and normal cartilage is detectable for a large number of genes.
*Conclusion:* With this newly developed isolation method high quality RNA can be obtained from low cellular tissue with high extracellular matrix component. This RNA is of suitable quality for subsequent cDNA microarray studies. These procedures can be applied to other difficult tumor material as well.

**076 *In vitro* Effectiveness of the Antineoplastic Drug Ecteinascidin-743 on Sensitive and Multidrug Resistant Osteosarcoma Cells**

Katia Scotlandi[1], Stefania Perdichizzi[1], Maria Cristina Manara[1], Massimo Serra[1], Glynn Faircloth[2], Maurizio D'Incalci[3], Piero Picci[1]
*[1]Istituti Ortopedici Rizzoli- Laboratorio di Ricerca Oncologica, [2]Pharma Mar USA, [3]Istituto Di Ricerche Farmacologiche Mario Negri Department of Oncology*

*Objective:* Ecteinascidin-743 (ET-743) is a highly promising antitumor agent isolated from the marine tunicate Ecteinascidia turbinate and is currently under phase II clinical investigation in Europe and the United States. Preclinical studies have shown that ET-743 is active against a variety of tumor cell lines *in vitro* and against several rodent tumors and human tumor xenografts *in vivo* at low concentrations. In this study the antitumor activity of this drug was evaluated against a panel of human osteosarcoma cell lines characterized by different drug responsiveness.
*Methods:* A panel of 13, P-glycoprotein negative, human osteosarcoma cell lines as well as 6 multidrug-resistant (MDR), P-glycoprotein overexpressing, variants and two methotrexate- resistant cell derivatives were analyzed *in vitro* to test the effectiveness of ET-743 and its mechanisms of action. The degree of sensitivity was expressed as the drug concentration resulting in 50% inhibition of growth (IC50) after 1 h or 96hr of continuous exposure to ET-743. Cell cycle analysis and Bromodeoxyuridine labeling index were obtained by flow cytometry after 24, 48 and 72 hr of drug exposure. Cell death induced by ET-743 was evaluated by Annexin-test and morphologic analysis of apoptotic nuclei.
*Results:* 12/13 osteosarcoma cell lines showed an IC50 value ranging from 0.3 to 1 nM after a long-term exposure. These concentrations are remarkably low, very well achievable in clinical conditions and indicate that osteosarcoma is particularly sensitive to ET-743. In comparable conditions, ET-743 was overall more active than doxorubicin, methotrexate and cisplatin, the three leader drugs in the treatment of osteosarcoma. Furthermore, this drug was found to be completely active against methotrexate-resistant cells and significantly overcome MDR. Cell lines showing up to 200-fold of resistance against doxorubicin, exhibit resistance levels to ET-743 lower than 10-fold. Long-term exposure produced a higher extent of inhibition than a one-hour exposure, especially on MDR cells. At IC50 doses, ET-743 appeared to have a cytostatic rather than a cytotoxic effect. Cells exposed to ET-743 progress through the cell cycle more slowly than untreated cells. No induction of apoptosis was observed at these concentrations.
*Conclusion:* ET-743 appears to be very effective against osteosarcoma cell lines, both against P-glycoprotein-negative and P-glycoprotein overexpressing cells. This is particularly relevant since P-glycoprotein is a major adverse prognostic factor in this neoplasm. Therefore, ET-743 should be considered in the treatment of osteosarcoma patients.

**080 Anchorage Independent Growth and Prolonged Survival of Primary Human Osteoblasts Over-Expressing the Met Receptors by Means of Transduction with Lentiviral Vectors**

N Coltella[1], S Patane'[1], S Avnet[3], M Olivero[1], E Vigna[2], L Naldini[2], N Baldini[3], M F Di Renzo[1], R Ferracini[1]
*[1]Laboratory of Cancer Genetics of the Institute for Cancer Research and Treatment (IRCC), [2]Laboratory of Gene Therapy of the Institute for Cancer Research and Treatment (IRCC), [3]Laboratory for Pathophysiology of Orthopaedic Implants, Istituti Ortopedici Rizzoli*

*Objective:* Osteoblast proliferation and differentiation is achieved through the integrated effects of several signalling molecules, which switch on receptor-mediated events and activate gene transcription. Each of the molecules controlling critical steps might play a role in the genesis of osteoblast-derived tumours, including receptor and non-receptor tyrosine kinases. Fragmentary data suggest that among these receptors, those of the MET family might also be involved in bone development and in osteoblast transformation. The MET oncogene-encoded tyrosine kinase receptor and its ligand Hepatocyte Growth Factor (HGF) ordinarily constitute a paracrine signalling system where cells of mesenchymal origin produce the ligand, which binds to receptors expressed on cells of epithelial origin. However, during mouse development HGF and met are both expressed in tissues of mesenchymal origin and regulate migration of myoblast precursors. We previously showed that the MET receptor is aberrantly over-expressed in a number of human osteosarcomas producing the ligand, suggesting that over-expression with or without autocrine activation of MET receptors might contribute to transformation of osteoprogenitor cells.
*Methods:* To demonstrate the transforming potential of the MET oncogene in human osteoblasts, first we constructed an ex-vivo model using primary 2D cultures of human osteoblasts with stable expression of the MET receptors by means of transduction with Lentiviral vectors. Bicistronic Lentiviral vectors carrying either wild-type or mutation-activated (METY1235D) MET cDNA followed by IRES (internal ribosomal entry site) and GFP-encoding sequences were used to transduce human osteoblasts. IRES sequences will ensure the concomitant expression of the MET receptor and the reporter protein, allowing the detection and the selection in vitro and in vivo of transduced cells. The human primary cultures transduced were from bone fragments and consisted of approximately 90% cells showing osteoblast phenotype. The latter was assessed with alkaline phosphatase assay and alizarine red staining of cells growing in differentiating medium.
*Results:* Propagated transduced osteoblasts showed a peculiar morphology and behaviour: cells were spindle-shaped and small, did not show cell contact inhibition in monolayer cultures and form colonies in soft agar. Different levels of MET expression were detectable in osteoblast clones and correlated with the ability of the cells to form colonies in agar. MET expressing osteoblasts grew for more than 30 passages in culture, corresponding to approximately 150 days. After 100 days the expression of the transgene declined but the level of MET receptor expression remained elevated. Most of the MET receptor stably over-expressed in long-term cultures was due to activation of the endogenous MET gene transcription.
*Conclusion:* These data show that over-expression of the MET oncogene in human primary osteoblast due either to transgene transduction or reactivation of endogenous MET expression confers to cells of mesenchymal origin the ability to grow in an anchorage independent fashion and to survive longer than the parental line. Further experiments will assess if the prolonged survival of osteoblasts depends on a block of apoptosis or to inactivation of regulated Rb control or to a telomer lengthening activity.

**086 Expression of Osteopontin and HGF/SF-Met in Adult Soft Tissue Sarcoma**

Stewart C. Rorke[1], Vivien H. Bramwell[1], Ann F. Chambers[1], Alan B. Tuck[2], Larry Stitt[3]
*[1]London Regional Cancer Centre, [2]London Health Sciences Centre, [3]University of Western Ontario*

*Objective:* (1) To determine the range of expression of osteopontin (OPN) (a secreted phosphoprotein associated with malignant transformation) and Met (an oncogene encoded transmembrane kinase) in adult soft tissue sarcoma (ASTS). (2) To determine if expression of these markers is associated with clinical outcome variables.
*Methods:* Thirty-three tissue samples from ASTS were obtained from a cohort of patients registered in the Canadian Soft Tissue Sarcoma Tumor Bank/Correlative Clinical Data Base. OPN and Met expression were determined by semiquantitative immunohistochemistry. Results were expressed as a total score derived from a combination of intensity (0-3) and proportion (0-5) of staining, for a potential total out of 8.
*Results:* Included in the cohort were 8 superficial and 27 deep tumours, with a median maximum diameter of 9 cm. There were 33 primary tumours and 1 local recurrence. Most common among a wide range of histologies were malignant fibrous histiocytoma (9), liposarcoma (7), and leiomyosarcoma (6). Number of patients with grades 1, 2, 3-4 were 5, 13, and 12 respectively (not available (N/A) in 3 patients). UICC clinical stage (at the time of sample submission):I(6), II (14), III (6), IV (3), N/A (4). Surgeries included: amputation (2), local marginal resection (22), and wide resection (9). Adjuvant chemotherapy and radiation was given in 4 and 15 patients respectively. Two patients received neoadjuvant chemotherapy. Median follow-up time was 40 months (range 2–84 months) and the 2-year overall survival was 69%. Mean OPN and Met scores were 2.8 and 1.4 respectively. Scores ranged from 0 to 7 in OPN and 0 to 6 in Met, with standard deviations of 2.2 and 2.0 respectively. With respect to OPN score, Spearman correlation coefficients for stage and grade were 0.479 (p=0.009) and 0.500 (p=0.005) respectively, indicating strong positive associations. Univariable Cox regression analysis showed that increasing OPN levels predict decreased disease-free survival (DFS) (Hazard ratio (HR) = 1.55, (95% CI:1.15-2.10), p=0.003). Similarly, OPN was significantly associated with decreased overall survival (OS)(HR=1.31, (95% CI: 1.006-1.714), p=0.045). Met scores were not significantly associated with outcome.
*Conclusion:* In this small retrospective analysis of an ASTS cohort: (1) Increased OPN expression (determined immunohistochemically) was associated with decreased DFS and OS. (2) Level of Met expression was not significantly associated with outcome. There are many potential pitfalls in analysis of marker expression in small inhomogeneous cohorts of patients, and these findings need to be explored in a larger series of ASTS cases.

**089 Fusion of the ALK Gene to the Clathrin Heavy Chain Gene, CLTC, in Inflammatory Myofibroblastic Tumor**

Julia A Bridge[1], Masahiko Kanamori[1], Zhigui Ma[2], D Ashley Hill[2], William Lydiatt[1], Man Yee Lui[3], Gisele WB Colleoni[3], Cristina R Antonescu[3], Marc Ladanyi[3], Stephan W Morris[2]
*[1]Departments of Pathology/Microbiology and/or Pediatrics and/or Orthopaedic Surgery and/or Otolaryngology, University of Nebraska Medical Center, [2]Departments of Pathology and/or Hematology/Oncology, St. Jude Children's Research Hospital, [3]Department of Pathology, Memorial Sloan-Kettering Cancer Center*

*Objective:* Recently, clonal mutations [fusion of the tropomyosin (TPM) N-terminal coiled-coil domains to the ALK C-terminal-kinase domain] have been detected in a subset of inflammatory myofibroblastic tumors (IMTs) supporting a neoplastic pathogenesis for this biologically controversial entity (*Am J Pathol* 2000; 157:377). The ALK gene is localized to chromosomal band 2p23 and the TPM3 and TPM4 genes are localized to 1q22-23 and 19p13.1 respectively. In the current study, cytogenetic analysis of an IMT revealed a 2;17 translocation [t(2;17)(p23;q23)]. Because 17q23 is not the chromosomal locus of either TPM3 or TPM4, our objective was to determine the ALK fusion gene partner in this case and in an additional IMT not exhibiting a TPM3-ALK or TPM4-ALK fusion gene transcript.
*Methods:* Case 1 was obtained from an IMT of the neck of a 3-year-old female and Case 2, an IMT of the pelvis of a 37-year-old male. Conventional cytogenetic analysis was performed on a sterile, representative tissue sample from Case 1. Material suitable for cytogenetic analysis was not available for Case 2. Fluorescence *in situ* hybridization (FISH) studies employing 2p23 (ALK) breakpoint spanning and flanking probes (Vysis, Downers Grove, IL) were executed on metaphase preparations of Case 1 and on cytologic touch preparations of both cases. Rapid amplification of cDNA ends (RACE) studies were performed on Case 1 using the 5' RACE system from Gibco BRL (Rockville, MD). The products were analyzed on agarose gels. Sharp product bands were subjected to direct sequencing, whereas non-discrete products were cloned and multiple independent clones were sequenced. Following RACE analyses, heminested reverse transcriptase – polymerase chain reaction (RT-PCR) studies were performed to confirm the presence of a CLTC-ALK fusion gene in both cases. The identified product bands were directly sequenced.
*Results:* Case 1 exhibited the following: 46,XX,t(2;17)(p23;q23), add(16)(q24)[5]/ 92,idem x2[1]/46,XX[4]. Case 1 metaphase cell FISH confirmed the presence of a 2;17 translocation involving the ALK locus. Cases 1 and 2 interphase cell FISH revealed a split of one set of the two-color probe signals, indicating a disruption of the 2p23 breakpoint (ALK) on one chromosome 2 homologue in 52% and 46% of the cells, respectively. 5' RACE of Case 1 revealed an ALK fusion with the clathrin heavy chain gene (CLTC) localized to 17q23. This CLTC-ALK fusion incorporates 1,634 residues of the 1,675-aa clathrin heavy chain. Heminested RT-PCR confirmed the presence of a CLTC-ALK fusion gene product in Case 1 and demonstrated the identical fusion product in Case 2. The ALK and CLTC breakpoints in these IMTs were identical to those recently reported in CLTC-ALK fusions in anaplastic large cell lymphoma (ALCL), (*Blood* 2000; 95:3204).

*Conclusion:* The CLTC-ALK fusion oncogene represents a novel mechanism of ALK activation in IMT and demonstrates that, similar to ALCL, the fusion partners of the ALK gene in IMT are diverse. ALK protein expression is an independent predictor of survival and serves as a useful biological marker of a specific disease entity within the spectrum of ALCL. Additional studies are warranted to determine whether ALK protein expression is likewise associated with specific clinicopathological traits in IMT.

**090 Evaluation of Ezrin, a Novel Determinant for Metastasis in Osteosarcoma: A Comparative Approach**

Chand Khanna[1], Seuli Bose[1], Sung-Hyeok Hong[1], Ryan Cassaday[1], Stephen Hewitt[2], Richard Gorlick[3], Lee Helman[1]
*[1]Pediatric Oncology Branch, National Cancer Institute, National Institutes of Health, [2]Laboratory of Pathology Tissue Array Research Project, National Cancer Institute, National Institutes of Health, [3]Memorial Sloan-Kettering*

*Objective:* To evaluate the importance and relevance of ezrin in the biology of osteosarcoma metastasis using a cross species comparative approach (murine, canine, and human). This approach allows tissues from children and pet dogs with naturally occurring osteosarcoma to add relevance to genomics data generated from our *in vitro* and *in vivo* metastasis models.
*Methods:* Using cDNA microarrays and a metastasis based methodology for array evaluation we have defined 11 genes (out of 3899 cDNAs) most likely to explain differences in the behavior of a high (K7M2) and low (K12) metastatic model of murine osteosarcoma. Ezrin, a gene not previously described in osteosarcoma, was selected for further evaluation based on its pivotal role in linking the actin cytoskeleton to the cell membrane. Evaluation of ezrin in the murine osteosarcoma models included Northern, Western, and immunocytochemistry. The function of ezrin was studied by generating K7M2 cells with low expression of ezrin through the stable transfection of these cells with a full-length antisense ezrin construct. Preliminary evaluation of ezrin function included transwell® matrigel invasion and heterotypic adhesion assays. The relevance of ezrin in osteosarcoma was examined in a canine osteosarcoma tissue array consisting of 75 canine osteosarcoma primary tumors and 11 pulmonary metastases. The expression of ezrin in human osteosarcoma was examined by immunohistochemistry.
*Results:* Differential expression of ezrin between K7M2 and K12 models by Northern was concordant with microarray (2.9:1). Evidence for differential post-transcriptional regulation came from Western analysis where an 8.0 fold increase in ezrin protein was seen in highly metastatic K7M2 compared to poorly metastatic K12. Immunostaining was consistent with Western results and supported greater membrane (active) localization of ezrin in the more aggressive K7M2 cells. Stable antisense ezrin transfection of the K7M2 cells resulted in clones with low (K7M2-AS1.42 and K7M2-AS1.56) and intermediate (K7M2-AS13) ezrin protein levels. Functional studies compared the antisense ezrin transfectants with an empty vector control (K7M2-EV) and the wild-type K7M2 and K12 cells. K7M2-AS1.42 and K7M2-AS1.56 had notably decreased invasiveness through matrigel compared to K7M2-AS13 and the K7M2-EV control ($p=0.06$ and $p=0.05$ respectively). K7M2-AS1.42, K7M2-AS1.56 and K7M2-AS13 had heterotypic adherence kinetics similar to the less metastatic K12 cells. Immunohistochemistry for ezrin using the canine osteosarcoma tissue array demonstrated high ezrin expression in 100%(11/11) of pulmonary metastases compared to 30% (42/70) of primary tumors ($p=0.0001$). Furthermore dogs without primary tumor ezrin had longer median survival than dogs with ezrin staining (Ezrin(-) 280 days vs Ezrin(+) 136 days; $p=0.10$). In frozen pediatric osteosarcoma ezrin expression was seen in 8/12 tissues by immunohistochemistry. High intensity staining was seen in 0/7 primary tumors and 2/5 pulmonary metastases.
*Conclusion:* This comparative approach has demonstrated the relevance and potential importance of ezrin in the biology of osteosarcoma metastasis. Preliminary results suggest a role of ezrin during cellular invasion and heterotypic adhesion. Ongoing efforts will define the role of ezrin *in vivo* and through all steps of the metastatic cascade. Staining of a recently constructed pediatric osteosarcoma tissue array has been completed and will define the relevance of ezrin in pediatric osteosarcoma.

**091 Chromosome 9 Alterations and P16 Expression in Central Chondrosarcomas**

J. V. Bovee[1], D. Federov[1], H. van Beerendonk[1], A. H. Taminiau[4], R. Sciot[2], M. Debiec-Rychter[3], A. M. Cleton-Jansen[1], P. C. Hogendoorn[1]
*[1]Department of Pathology, Leiden University Medical Center, [2]Department of Pathology, Leuven University, [3]Center for Human Genetics, Leuven University, [4]Department of Orthopedic Surgery, Leiden University Medical Center*

*Objective:* Chondrosarcomas are characterized by neoplastic growth of cartilage forming tumor cells. The majority (75%) arise centrally, in the medullar cavity, while a minority develop peripherally secondary to an osteochondroma. We previously investigated DNA-ploidy and loss of heterozygosity (LOH) at loci harboring the EXT-genes (implicated in hereditary multiple exostoses), the EXT-like genes, and at 9p21, 13q14, 17p13 and chromosome 10 in 12 central chondrosarcomas. Only 3 cases exhibited LOH, with 9p21 involved in all three. At 9p21 the p16 tumour suppressor gene is located. Our goal was to further investigate this chromosomal region and the expression of the candidate gene p16.
*Methods:* LOH analysis was performed in the region of p16 on 38 additional cases. Cytogenetic analysis was performed on 16 central chondrosarcomas. p16 immunohistochemistry was performed on formalin-fixed paraffin-embedded tissue of 84 cases to estimate the effect of 9p21 alterations on p16 protein expression.
*Results:* Of 38 central chondrosarcomas 12 (32%) revealed LOH in the 9p21 region. Seven central chondrosarcomas demonstrated an abnormal karyotype, 5 of which involved chromosome 9. Three central tumours showed involvement of the 9p12-22 region including t(9;10)(p22;q22), add(9)(p21) and add(9)(p12). For two of them paraffin blocks were available revealing absence of p16 protein expression. Two tumours with -9 and del(9)(q12) demonstrated p16 protein expression. In 3 chondrosarcomas demonstrating LOH at 9p21, p16 protein expression was absent, while in 6 out of 9 central chondrosarcomas without LOH at 9p21, p16 protein expression could be demonstrated. In general loss of expression of p16 by immunohistochemistry was found in 18 % of the cases.
*Conclusion:* The involvement of genes located at chromosome 9, especially the 9p12-22 region, is suggested both by the LOH results, p16 immunohistochemistry as well as the cytogenetic studies. Since 9p21 alterations are associated with the absence of p16 protein expression, this suggests an important role for the p16 tumour suppressor gene in the development of central chondrosarcomas.

**100 Classification of Gene Expression Profiles in Adult Soft Tissue Sarcoma Using Oligonucleotide Array Analysis**

Neil H Segal, Robert G Maki, Alex Smith, Elyn Riedel, Katherine S Panageas, Cristina R Antonescu, Jonathan J Lewis, Murray F Brennan, Alan N Houghton, Carlos Cordon-Cardo
*Memorial Sloan-Kettering Cancer Center, 1275 York Avenue*

*Objective:* Adult soft tissue sarcoma (STS) represents a diverse group of neoplastic diseases that are grouped together because of shared biological characteristics and clinical responses [1]. This study was undertaken to identify the differential gene expression profile obtained from various histological categories of STS. Large scale gene expression data could be used to validate the current classification system for STS, investigate an alternative gene expression based classification system, and furthermore identify genes that differentiate between tumors of varying clinical outcome. Gene expression profiles may offer more information than classic morphology and provide an alternative to morphology-based tumor classification systems.

*Methods:* Total RNA was isolated from 30 cases of high grade STS, including leiomyosarcoma, fibrosarcoma, liposarcoma, synovial sarcoma, GIST, MFH and clear cell sarcoma. cRNA was prepared according to the Affymetrix® protocol and hybridized to the U95A GeneChip® array. An average difference (AD) value was generated that corresponds to the level of gene expression. Expression data was scaled to 2500 using the 96% mean-centered method. Absent calls and negative values were set to an overall average background value. Gene lists by tumor type were generated by ranking of F-statistic values. Multi-dimensional scaling was also applied to the original data in an unsupervised analysis to demonstrate inherent similarity of STS tumors.

*Results:* Expression data was determined for ~12 500 genes previously reported in terms of function or disease association (Affymetrix®). Individual tumors showed distinct patterns of gene expression. The top 100 genes were selected by rank of F-statistic to discriminate the test group of STS. 21 genes were shown to discriminate synovial sarcoma. PRAME, a cancer-testis antigen recognized by autologous T cells in melanoma [2], was shown to be expressed in synovial sarcoma and to a lesser extent in fibrosarcoma. 40 genes were shown to discriminate GIST, including c-kit. 39 genes were shown to discriminate clear cell sarcoma. Using unsupervised multidimensional scaling, across ~10 500 genes, synovial sarcoma, GIST and clear cell sarcomas emerge as distinct clusters. The remaining specimens did not appear to separate into histological or distinct genetic groups (figure).

*Conclusion:* We identified several potentially important genes in the diagnosis and biology of STS. We have shown that synovial sarcoma, GIST and clear cell sarcoma are genetically distinct within themselves and relative to the more homogeneous group of high grade leiomyosarcoma, fibrosarcoma and MFH. The liposarcoma group is as yet inconclusive, being comprised of dedifferentiated and pleomorphic liposarcomas. MFH and fibrosarcomas grouped together. These data indicate that MFH may represent pleomorphic fibroblastic tumors rather than tumors with a distinct histiocytic histogenesis. GISTs, previously considered as gastrointestinal leiomyosarcomas, stand out as a separate group characterized by abnormalities in c-kit. Research in progress aims to identify gene lists that may discriminate between the latter group of tumors, and to characterize selected transcripts that may provide insight into the pathology and clinical behavior of STS. 1. Mann, G.B. *et al. Aust N Z J Surg*, 1999; 69(5): 336-43. 2. Ikeda, H., *et al. Immunity*, 1997; 6(2): 199-208.

### 117 Impact of SYT-SSX Fusion Type on Clinical Behavior of Synovial Sarcoma. A Multi-Institutional Study of 243 Patients

Marc Ladanyi[1], Cristina R. Antonescu[1], Murray F. Brennan[1], Julia A. Bridge[2], Frederic G. Barr[3], Janet Shipley[4], Colin S. Cooper[4], Cyril Fisher[4], Björn Skytting[5], Olle Larsson[5]
*[1]Memorial Sloan-Kettering Cancer Center, [2]University of Nebraska Medical Center, [3]Hospital of the University of Pennsylvania, [4]Institute for Cancer Research and The Royal Marsden NHS Trust, [5]Stockholm Söder Hospital and Karolinska Hospital*

*Objective:* Synovial sarcomas consistently contain a specific t(X;18)(p11;q11), which represents either of two gene fusions, SYT-SSX1 or SYT-SSX2. Previous studies have suggested that patients with SYT-SSX2 tumors do better than those with SYT-SSX1 tumors, but the study groups were relatively small.

*Methods:* To address this issue more definitively, we collected data on SYT-SSX fusion type, pathology, and clinical course in a retrospective multi-institutional study of 243 patients (of whom 44% were under age 30 – age range 6-82) with synovial sarcoma, of which 89% were localized at presentation and 67% were extremity primaries.

*Results:* SYT-SSX1 and SYT-SSX2 fusions were detected in 147 tumors (61%) and 91 tumors (37%), respectively. There were 180 (74%) monophasic and 61 (25%) biphasic tumors. The previously observed association of fusion type and histology was confirmed in this series (n=236; $p<0.001$). Of 236 cases with data for both fusion type and histologic type, there were only 3 SYT-SSX2+ biphasic tumors. There was also a statistically significant association of fusion type and patient sex (n=232; p=0.03); the male-female ratio of SYT-SSX1 cases was 1:1, whereas for SYT-SSX2 cases, it was close to 1:2. There was a trend for patients with SYT-SSX1 + tumors to present more often with metastatic disease (n=231; p=0.05). There was no significant association of fusion type with patient age and size or site (axial vs peripheral) of the primary tumor. Median and 5 year overall survivals for the SYT-SSX1 and SYT-SSX2 groups were 6.1 years and 53%, and 13.7 years and 73%, respectively. Overall survival was significantly better among SYT-SSX2+ cases (n=228; p=0.03), among cases localized at diagnosis (n=231; $p<0.0001$), and among patients with primary tumors less than 5 cm in greatest dimension (n=200; p=0.01). Age, sex, histologic type, and axial vs peripheral primary site had no impact on overall survival. The impact of fusion type on survival remained significant when stratified for primary tumor size (n=198; p=0.03) but was no longer significant when stratified for disease status at presentation (n=226; p= 0.16). Cox regression identified disease status ($p<0.0001$) and primary tumor size (p=0.04) as the only factors independently predictive of overall survival in the subset of 160 patients with information on all factors. Within the subset of patients with localized disease at diagnosis (n=202), the median and 5-year survival for the SYT-SSX1 and the SYT-SSX2 groups were 9.2 years and 61%, vs 13.7 years and 77%, respectively. Patients whose tumors contained the SYT-SSX2 fusion (n=202, p=0.08) or were smaller (n=174, p=0.12) showed a trend toward better survival by log rank test, whereas tumor histology had no impact (p=0.8). By Cox regression analysis considering all factors, SYT-SSX fusion type emerged as the only independent significant factor (p=0.044) for overall survival within the subset of 133 patients with localized disease at diagnosis who had information on all factors.

*Conclusion:* Overall, SYT-SSX fusion type appears to exert part of its impact on prognosis prior to presentation, through its association with stage at presentation, which remains the strongest prognostic factor in all patients.

### 122 Comparison of P53 Mutations in Localized and Metastatic Osteosarcoma

Jay S Wunder[1], Nalan Gokgoz[1], Sasha Eskandarian[1], Shelley B Bull[1], Aileen M Davis[1], Robert Parkes[1], Chris P Beauchamp[2], Ernest U Conrad[3], Robert J Grimer[4], D Chas Mangham[4]
*[1]Samuel Lunenfeld Research Institute, Mount Sinai Hospital, [2]Mayo Clinic, [3]University of Washington Medical Center, [4]Royal Orthopaedic Hospital, [5]Memorial Sloan Kettering Cancer Center*

*Objective:* In many cancers, tumors harboring mutations of the p53 gene have a more aggressive clinical course or are more likely to be from advanced disease. To address the role of p53 mutations in osteosarcoma development and progression we analyzed 247 primary localized osteosarcomas and 25 osteosarcomas that were metastatic at diagnosis. The group included 27 matched biopsy-resection and 21 biopsy-metastasis paired specimens.

*Methods:* Tumor specimens and corresponding clinical data were obtained from 272 patients with biopsy proven high-grade osteosarcoma of the extremity from six tertiary care institutions. The nature and location of p53 mutations (exons 4 through 10) were examined by PCR-SSCP (single strand conformation polymorphism), confirmed by direct DNA sequencing, and compared with clinicopathologic factors identifiable at the time of diagnosis. The prognostic significance of p53 mutation status was investigated in a cohort of 202 patients with classical osteosarcoma who were treated with chemotherapy and local tumor resection and followed prospectively. Survival analysis of p53 gene status was by the log-rank test and Cox proportional hazards model.
*Results:* In the entire group of 272 patients, the overall frequency of p53 mutations was 22% (60/272) with 13 of the 60 mutations located in exons 4 or 10. A similar proportion of localized osteosarcomas had alterations of the p53 gene (55/247, 22.3%) compared to tumors from patients with metastases at diagnosis (5/25, 20%; p=0.96). Tumors from patients with localized osteosarcomas and those with metastases at diagnosis also exhibited equal proportions of missense (32/247, 13% vs. 3/25, 12%) and nonsense (23/247, 9% vs. 2/25, 8%) mutations respectively. Patients with p53 missense mutations were older than those with nonsense alterations or a wild-type gene (p=0.01). Tumor site (p=0.006) and tumor size (p=0.002) were the only factors associated with systemic disease status at diagnosis, but neither was related to p53 status. Examination of paired biopsy-resection and biopsy-metastasis specimens revealed that the p53 status was concordant between the biopsy and later tumor specimens in all cases. In the prospective cohort of 202 patients with localized osteosarcoma, there was no significant association between the presence of a p53 mutation and development of systemic disease recurrence by either univariate (p=0.18) or multivariate (p=0.16) analysis.
*Conclusion:* P53 mutation status was concordant for all paired tumor specimens, and did not differentiate between patients presenting with a localized osteosarcoma and those with metastases at diagnosis. These results indicate that p53 mutations are not late events in osteosarcoma tumor progression as they are evident before the development of metastases. Even for patients presenting with a localized osteosarcoma, p53 status was not a predictor of disease outcome. New molecular prognostic features of osteosarcoma are needed to improve patient stratification and treatment.

**123 Gene Expression Profiles of Low Grade and High Grade Malignant Fibro Histiocytomas Using Microarray Analysis**

Nalan Gokgoz[1], Jay S Wunder[1], Sasha Eskandarian[1], Chao Lu[2], Cecilia Cotton[1], Shelley B Bull[1], Robert S Bell[1], Irene L Andrulis[1]
[1]*Fred A. Litwin Center for Cancer Genetics, Samuel Lunenfeld Research Institute, Mount Sinai Hospital,* [2]*Ontario Cancer Institute, Princess Margaret Hospital,*

*Objective:* Malignant Fibrous Histiocytomas (MFH) are group of malignant mesenchymal tumors that present a dilemma for clinical management. Current staging systems, based on morphological tumor characteristics cannot accurately classify or predict an individual patient's risk for eventual metastases. We hypothesize that characterization of gene expression patterns of MFH will improve classification of these tumors. To identify clinically meaningful patterns of gene expression in MFH we are taking advantage of a soft tissue sarcoma tumor bank and a high-throughput microarray technology which is capable of profiling gene expression patterns of tens of thousand genes in a single experiment.
*Methods:* Tumor specimens were obtained from 37 patients with MFH who did not receive preoperative chemotherapy or radiotherapy. Total RNA was extracted with the RNeasy purification kit (Qiagen). 5μg of tumor and reference RNA were labeled with Cy3-dCTP and Cy5-dCTP fluorescent tags respectively, using an indirect labeling method and hybridized simultaneously to 19200 cDNA microarray chips. Reproducibility experiments were performed for each tumor/reference pair, in which the tumor and reference sample were re-assessed using the reciprocal fluorescent tag. The standard reference sample (i.e. control) consisted of a pool of RNA from 11 different tumor cell lines. Following hybridization, arrays were scanned using an Axon scanner and spots were quantitated with GenePix 3.0 analysis software.
*Results:* Before using the limited RNA from these specimens, we performed pilot studies to evaluate the feasibility of the technology on a larger scale using cDNA microarrays containing 1700 and 19200 sequence verified human cDNAs. We performed 10 self test experiments in which each pool of control RNA was split, labeled by Cy3 and Cy5 and simultaneously hybridized to the same 1700 cDNA array. The analysis of scatter plots for these experiments showed the consistency of the labeling and hybridization procedures, as evidenced by high correlation coefficients (R2 = 0.92-0.97). We also analyzed 5 MFH cases using 1700 cDNA chips. To allow for comparison of results between tumors each tumor was analyzed using the same control sample. Background subtracted signal intensities between the two fluorescent images were normalized by applying the median of Cy3/Cy5 log ratio as a normalization factor overall and by subarray for each microarray experiment. Comparison of the data across 10 experiments for 5 tumors showed that 12 genes had consistently high ratios and 17 genes had consistently low ratios. We further investigated 7 low grade and 8 high grade MFH tumors by using arrays with 19200 genes. Biostatistical modeling is being used to detect clusters of genes that may distinguish MFH tumors.
*Conclusion:* We have been able to devise a system that may distinguish the variation in gene expression of 19200 genes in low grade and high grade MFH tumors. Statistical approaches will enable us to detect sets of genes that are differentially expressed in high versus low grade tumors. Further evaluation will allow us to determine their potential use in differential diagnosis and early detection of MFH.

**127 Enchondromatosis Caused by a Mutant Type I PTH/PTHrP Receptor**

Sevan Hopyan[1], Nalan Gokgoz[1], Raymond Poon[2], Robert S. Bell[1], William G. Cole[2], Irene L. Andrulis[1], Benjamin A. Alman[2], Jay S. Wunder[1]
[1]*Musculoskeletal Oncology Unit and Program in Molecular Biology and Cancer, Mount Sinai Hospital,* [2]*Program in Developmental Biology, The Hospital for Sick Children*

*Objective:* Enchondromas are common benign cartilage tumours. When they occur in multiple locations in enchondromatosis (Ollier's disease), the risk of skeletal deformity and of malignant change to chondrosarcoma is high. Enchondromas are usually in close proximity to, or in continuity with, growth plate cartilage. Consequently, they may result from abnormal regulation of proliferation and terminal differentiation of chondrocytes in the adjoining growth plate. In normal growth plates, differentiation of proliferating chondrocytes to post-mitotic hypertrophic chondrocytes is regulated in part by a tightly coupled signalling relay involving Indian hedgehog (IHH) Parathyroid hormone related protein (PTHrP). We speculated that inappropriate regulation of the IHH-PTHrP pathway contributes to the genesis of enchondromas.
*Methods:* We utilized semiquantitative RT-PCR and Western blot analysis to test for expression of IHH-PTHrP pathway members, and a short term primary cartilage tumour explant culture system to test the functional effects of Hedgehog and PTHrP agonists and antagonist in vitro. Proliferation was assessed by tritiated thymidine uptake, and differentiation by type X collagen expression level (an exclusive product of hypertrophic chondrocytes). Single strand conformation polymorphism analysis and manual sequencing were used for mutational screening. *In vitro* Cyclic adenosine monophosphate (cAMP) and Inositol triphosphate (IP3) assays were performed using wild type (WT) and mutant constructs generated via site-directed mutagenesis, which were transiently transfected into COS-7 cells and embryonic stem cells lacking native Type 1 PTH/PTHrP receptor (PTHR1). Transgenic mice were generated by pronuclear microinjection of WT or mutant PTHR1 cDNA flanked by the regulatory elements of Type II collagen (ColII) for expression in cartilage. Genomic DNA was extracted from tails and screened by Southern blot for integration of the transgene. Paraffin embedded sections from transgenic mice were used for immunohistochemistry, safranin-O histology and tartrate resistant acid phosphatase (TRAP) staining.

*Results:* We showed that key IHH-PTHrP pathway members are expressed in enchondromas and chondrosarcomas. The IHH and PTHrP signalling pathways were functional, but the feedback loop regulating IHH was dysregulated in these lesions. We identified a mutant PTHR1 in two patients with enchondromatosis. This mutant lowered baseline cAMP level and abolished IP3 accumulation in vitro. Expression of the mutant, but not WT, PTHR1 in the growth plates of transgenic mice resulted in the appearance of multiple enchondromas. These enchondromas were likely caused by abnormal proliferation and not abnormal resorption, since growth plate zonal architecture was altered, but the number of TRAP positive cells, which resorb the growth plate, were not.

*Conclusion:* These data suggest that enchondromas can arise due to abnormal growth plate development. In particular, some cases of enchondromatosis are likely caused by a mutant PTHR1. The persistence of growth plate tissue in the form of enchondromas beyond adolescence, when growth plate tissue has normally disappeared in humans, may allow accumulation of secondary genetic events which cause chondrosarcoma to arise within a preexisting enchondroma. Agents that block IHH-PTHrP signalling might be of therapeutic benefit in preventing the deleterious consequences of enchondromas.

